# Self-assembled triptolide prodrug nanovesicles loading with ginsenoside Rg3 for double-targeted therapy of pancreatic cancer

**DOI:** 10.1016/j.mtbio.2025.102004

**Published:** 2025-06-18

**Authors:** Jiaxing Wang, Jingru Cui, Yujie Chen, Huijie Zhou, Xiaofang Li, Xiangxiang Wu, Rongyi Zhou, Huahui Zeng

**Affiliations:** a Academy of Chinese Medicine Sciences, Henan University of Chinese Medicine, Zhengzhou, 450046, China; b Collaborative Innovation Center of Research and Development on the Whole Industry Chain of Yu-Yao, Henan Province, Henan University of Chinese Medicine, Zhengzhou, 450046, China; c Pediatric Medicine Department, The First Affiliated Hospital of Henan University of Chinese Medicine, Zhengzhou, 450003, China

**Keywords:** Ginsenoside Rg3, Triptolide, Nanovesicle, Prodrug, Pancreatic cancer

## Abstract

Triptolide (TP), derived from the herb Tripterygium wilfordii, has a highly potent antitumor effect, but its poor water solubility and high toxicity hinder its clinical use. Here, a novel triptolide prodrug (TP-PEG-SS) was synthesized by conjugating TP and stachydrine (SS) with polyethylene glycol (PEG), which endowed TP with high water solubility, the capability to target tumor mitochondria, significant antitumor efficacy and low toxicity. Subsequently, TP-PEG-SS was self-assembled with ginsenoside Rg3 and lecithin to form nanovesicles (NVs). The NVs exhibited double-targeted performance for actively targeting tumor mitochondria via electrostatic interaction and entering M2 macrophage via glucose transporter GLUT-1, thereby greatly inhibiting the tumor cell growth by triggering apoptosis of tumor and polarization of M1 macrophage. In Pan02 tumor-bearing mice, the NVs were selectively accumulated in the tumor regions and improved the immunosuppressive tumor microenvironment, thereby exerting a more potent synergistic antitumor effect of both Rg3 and TP, as well as less systemic toxicity than free TP. Consequently, the NVs is a promising antitumor nanovesicle with double-targeted capability, which may enhance the clinical applicability of TP.

## Graphical abstract

**Figure:**
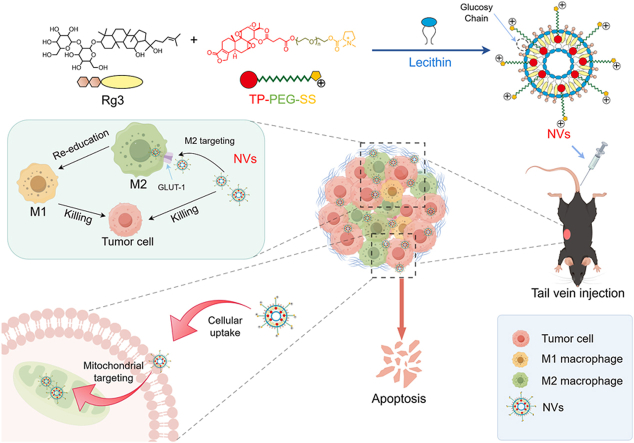

## 1 Introduction

Pancreatic cancer, a malignant tumor arising from pancreatic duct epithelium and acinar cells, is generally treated with radiotherapy, chemotherapy and surgery. In clinical practice, the challenges associated with treatment of pancreatic cancer include the lack of early diagnosis, poor prognosis, early metastasis, and multidrug resistance. However, radiotherapy and chemotherapy have greater adverse effects and systemic toxicity. After surgical resection, there is a significant probability of recurrence and less than 5 % five-year survival [1,2]. It is therefore imperative that a novel strategy for treatment of pancreatic cancer is developed, which has minimal side effects and high therapeutic effect.

Triptolide (TP), an epoxide diterpenoid derived from Tripterygium wilfordii, is an effective traditional Chinese medicine (TCM) ingredient with antitumor activity [3]. Recently, TP has drawn extensive attention due to its highly efficient antitumor effects [4,5]. In particular, TP exhibits higher anti-pancreatic cancer activity than gemcitabine, paclitaxel, cisplatin and adriamycin in many studies [6]. Previous researches reported that TP can induce pancreatic cancer cell apoptosis via the mitochondria pathway [[7], [8], [9]]. However, the high toxicity [10], poor water solubility [11], lack of tumor selectivity, and low bioavailability limit its clinical development as an antitumor agent [12]. Currently, we have carried out a series of chemical modifications on TP for improving its physicochemical properties and bioactivities. The modified triptolide derivatives, such as TP-CMCS, TP-CSO, and CCTP [5,13], exhibited higher solubility (5–15 mg/mL) in water, lower systemic toxicity, and higher bioavailability as compared with the free TP. Here, we designed and synthetized a novel TP-PEG-SS conjugate containing TP, polyethylene glycol (PEG), and stachydrine (SS), which endowed TP with the capacity to target tumor mitochondria through the SS cationic moiety. We previously reported that the stachydrine and its derivatives can actively penetrate the mitochondrial membrane and induce tumor cell apoptosis, due to the electrostatic interaction between the negatively charged mitochondria and the positively charged stachydrine derivatives [7,14]. It is anticipated that the conjugates will reduce the systemic toxicity of TP while increasing its water solubility and anticancer activity.

Ginsenoside Rg3 (Rg3) is the first clinical anticancer TCM ingredient derived from ginseng, which is usually used in conjunction with chemotherapy to greatly increase curative effect while reducing adverse effects. Rg3 can induce mitochondria-dependent apoptosis of lung cancer cells and significantly inhibit cell proliferation [15]. Additionally, Rg3 can promote paclitaxel-induced apoptosis and cytotoxicity via inhibiting NF-κB activation and increasing the ratio of Bax/Bcl-2 on triple-negative breast cancer [16]. The combining Rg3 and gemcitabine induced apoptosis of pancreatic cancer cells through CASC2/PTEN signaling [17]. Our previous studies showed that Rg3 significantly enhanced cytotoxicity and apoptosis of celastrol on pancreatic cancer cells, thereby enhancing antitumor efficacy [18]. By this token, Rg3 might promote the TP-induced mitochondria apoptosis of pancreatic cancer cells. Furthermore, Rg3 has the capability to repolarize pro-tumor M2 macrophages to anti-tumor M1 phenotype, thereby remodeling the tumor microenvironment and enhancing the antitumor efficacy [19,20]. Rg3 has a steroid structure, which can substitute cholesterol as a membrane material to provide the fluidity and stability for liposomes. In addition, two glucosyl residues of Rg3 are the natural substrates of glucose transporter protein type 1 (Glut-1) [20,21]. The M2-like tumor-associated macrophages (M2-like TAMs) overexpress Glut-1 [22] which mediates glucose transport and enhances the uptake of intratumoral glucose by M2-like TAMs. Thus, Glut-1 is a perfect receptor of Rg3, enabling substances with a structure of glucose to specifically target M2-like TAMs.

**Scheme 1:**
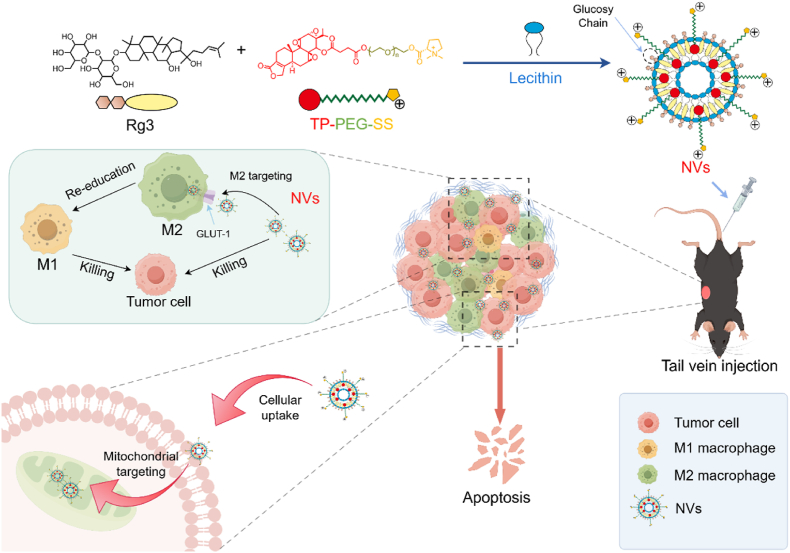
Schematic diagram of the preparation process and synergistic anticancer of nanovesicles (NVs). The NVs actively accumulate in the tumor regions by double targeting both tumor mitochondria and M2 macrophage, ultimately leading to a synergistic anticancer via triggering apoptosis of tumor and polarization of M2 macrophage.

Nanoformulation has currently become an important tool for the tumor cell or organelle-targeted delivery of drugs [7,23,24]. Nanoformulation offers many advantages, including the potentials for reducing drug dosage and frequency of administration, and improving its cell accumulation and bioavailability [[25], [26], [27]]. Rg3-PTX-LPs containing Rg3 and paclitaxel could actively distribute into breast cancer cells and tumor microenvironment by the recognition of GLUT-1, which achieved a high antitumor effect through apoptosis pathway [28]. Here, TP-PEG-SS, Rg3 and lecithin (PC) were employed in the preparation of nanovesicles (Rg3/TP-PEG-SS NVs, i.e. NVs), which didn't contain cholesterol and could specifically target tumor sites via the mitochondrial pathway [7] and Glut-1 pathway. Thus, the NVs had double-targeted performance for both tumor mitochondria and M2 macrophage (Scheme 1). The NVs were preliminarily confirmed to improve the synergistic antitumor effect of Rg3 and TP, and weaken the systemic toxicity of TP *in vivo and in vitro*.

## 2 Experimental materials and methods

### 2.1 Experimental materials

Triptolide was procured from Xi'an Haoxuan Biotechnology Co., Ltd (Xi'an, China). Ginsenoside Rg3 was procured from Chengdu Mansite Biotechnology Co., Ltd. Phosphatidylcholine and Coumarin 6 were obtained from Aladdin. Fetal bovine serum (FBS) was procured from Invintech. Other reagents, unless specified otherwise, were obtained from Tianjin Fuyu Fine Chemical Co., Ltd and Solarbio Science & Technology Co., Ltd. (Beijing, China). The mouse pancreatic cancer cell line Pan02 was obtained from Wuhan Pricella Biotechnology Co., Ltd. C57BL/6J mice (male, 14 g) and BALB/c nude mice (male, 14 g) were sourced from Beijing Huafukang Biotechnology Co. Ltd. (Production License No. SCXK(Beijing)2019-0008).

### 2.2 Preparation and characterization of TP-PEG-SS conjugate

A solution of 300 mg of NH_2_-PEG-NH-Boc (2 KDa) in 5 mL of DMF was prepared, followed by the addition of stachydrine (1.0 equiv.), PyBOP reagent (2.0 equiv.) and triethylamine (3.0 equiv.). Following a period of stirring at room temperature for 0.5 h, to precipitate the reaction solution, it was concentrated under reduced pressure and poured into ice ether. The products were collected by centrifuge and dried under vacuum to obtain stachydrine-PEG-NH-Boc.

In a dichloromethane solution, 200 mg of the above products was dissolved before adding 200 L of trifluoroacetic acid, followed by 0.5 h of reaction in an ice bath. An ice ether solution was added to the concentrated reaction solution to precipitate under reduced pressure. Stachydrine-PEG-NH_2_ was obtained by centrifuging and drying the products under vacuum.

10 mg of triptolide was dissolved in 400 μL of pyridine, added succinic anhydride (10.0 equiv.) and DMAP (0.1 equiv.). Following an overnight stirring period at room temperature, dichloromethane was introduced to the reaction solution. This was then washed in turn with saturated salt water, citrate water and salt water, each wash occurring three times. In order to precipitate the reaction solution, it was concentrated under pressure and poured into a large amount of ice ether. Triptolide-COOH was obtained by centrifuging the products and drying them under vacuum.

200 mg of stachydrine-PEG-NH_2_ was dissolved in 5 mL of DMF, added triptolide-COOH (1.0 equiv.), PyBOP reagent (2.0 equiv.) and triethylamine (3.0 equiv.). After stirring at room temperature for 0.5 h, a large amount of ice ether was used to precipitate the reaction solution under reduced pressure. The products were collected by centrifuge and dried under vacuum to obtain triptolide-PEG-stachydrine (TP-PEG-SS).

All prepared products underwent characterization using fourier-transform infrared spectroscopy (FT-IR, Nicolet iS50, Thermo Scientific), nuclear magnetic resonance spectrometer (^1^H NMR, 500 MHz, AV500 + BH0055, Bruker) and X-ray Diffraction (XRD) (D2 PHASER, Bruker Corporation, Karlsruhe, Germany) as previously described [5].

### 2.3 Preparation and characterization of nanovesicles (NVs)

The thin film dispersion method was used to prepare NVs. In brief, TP-PEG-SS, Rg3 and PC at a certain weight ratio were added into a round-bottom flask containing anhydrous methanol, and stirred continuously for 3 h. After the removal of the methanol using rotary evaporator, the thin film on the flask wall was dried under a vacuum drying oven. The ultrapure water was added into the flask and stirred continuously for 12 h, after which ultrasonic-assisted dispersion was performed for a further 5 min and extrusion was conducted through a 0.2 μm polycarbonate membrane in order to obtain the nanovesicles (Rg3/TP-PEG-SS NVs, i.e. NVs). The tumor mitochondria-targeted and M2 macrophage-targeted coumarin or DID nanovesicles (C6-SS NVs or DID-SS NVs containing Rg3 and TP-PEG-SS) and coumarin or DID nanoparticles (C6 NVs or DID NVs, without Rg3 and TP-PEG-SS) were prepared using the above method.

The particle size, polydispersity index (PDI), and zeta potential were detected by a Zetasizer Nano ZS particle analyzer (DLS) (NanoBrook 90^+^, Brookhaven, US). The morphology was observed using transmission electron microscopy (TEM) (JEM-1400, Japan) [7].

### 2.4 Encapsulation efficiency and drug loading capacity

After the preparation of NVs, a given amount of the lyophilized NVs was dissolved in methanol under sonication. The high-performance liquid chromatography (HPLC, Shimadzu, Japan) with a C18 column was used to measure the encapsulation efficiency (EE) and drug loading (DL) of NVs, as previously described [7]. The column eluant was acetonitrile-H_2_O (33:67 vol%) at a flow rate of 1.0 mL/min.

### 2.5 *In vitro* stability testing

The NVs were incubated with water, phosphate-buffered saline (PBS), fetal bovine serum (FBS), and complete culture medium (DMEM+10 % FBS) at 4 °C or 37 °C. The changes in particle size, PDI, and zeta potential were measured at various time periods.

### 2.6 *In vitro* drug release

Under 37 °C, NVs solution was poured into a dialysis bag (cut-off: 1 kDa), and immersed in PBS (pH 7.4) containing Tween 80 (0.5 %, v/v). The dialysates were withdrawn and then replenished with the equal volume of fresh PBS at predetermined intervals. The content of released drugs was detected by HPLC.

### 2.7 Cell culture and mouse husbandry

The Pan02 cells were cultured in DMEM high-glucose medium (1 % penicillin-streptomycin) containing 10 % FBS under the condition of 5 % CO_2_ at 37 °C in accordance with standard protocols. C57BL/6J mice or BALB/c nude mice were maintained under standard housing conditions (in a light-dark cycle environment at 25 ± 1 °C) and provided with fresh water and food. All animal studies were conducted in accordance with the guidelines of the Institute's Animal Care and Use Committee, and approved by the Experimental Animal Welfare Ethics Committee of Henan University of Chinese Medicine (Approval ID: IACUC-202308023).

### 2.8 Cellular uptake assay

Pan02 cells in a confocal culture dish (2 × 10^4^ cells/dish) were incubated with free C6, C6 NVs and C6-SS NVs for 1 h in the darkness, respectively. After incubation, the cells were washed thrice with sterile PBS and incubated with the Mito-Tracker Red CMXRos (Beyotime Biotechnology, Shanghai) for 20 min in accordance with the instructions. After the discard of the working solution, the cells were washed thrice with HBSS and incubated for another 20 min with Hoechst 33342. After the nuclear staining, the cells were immediately observed under a laser confocal microscope (CLSM) (Leica STELLARIS 5, Germany).

RAW264.7 cells were seeded in plates (2 × 10^5^ cells/well) for 24 h. The cells were subsequently induced to M1 macrophages using IFN-γ (2.5 ng/mL) and lipopolysaccharide (LPS, 200 ng/mL), and M2 macrophages using IL-4(10 ng/mL). The macrophage phenotypes were verified by qPCR to detect the mRNA expression levels of iNOS in M1 macrophages and Arg-1 in M2 macrophages, respectively. After washing with PBS, the cells were incubated with PBS, C6 NVs and C6-SS NVs for 1, 3 and 6 h, followed by evaluation using flow cytometry.

### 2.9 Cell viability and synergistic antitumor effect

A 96-well plate was seeded with Pan02 cells (approximately 5000 cells per well) and cultured for 24 h. After a 24-h incubation with different drugs, the cell viability was assessed using the CCK-8 assay, and the half inhibition concentration (IC_50_) and combination index (CI) were calculated as previously described [7].

Moreover, another 96-well plate was seeded with M1 or M2 macrophages (approximately 5000 cells per well). After a 24-h incubation with NVs, cell viability was assessed using the CCK-8 assay.

### 2.10 Cell apoptosis

Pan02 cells were incubated with TP (30 ng/mL), TP-PEG-SS, Rg3 and NVs (30 ng TP/mL eqv.) for 24 h. After being collected, the cells were suspended in 500 μL PBS. Subsequently, the cells were incubated with 5 μL of FITC-Annexin and 5 μL of PI for 15 min at room temperature in the dark. The flow cytometer (Beckman Coulter, USA) was used to measure the apoptosis rate.

### 2.11 *In vitro* M2 phenotype macrophage re-education

The M2 macrophages induced from RAW264.7 cells were incubated with PBS, TP-PEG-SS, Rg3, and NVs for 24 h. After their collection and resuspension in 100 μL of Cell Staining Buffer, the cells were incubated with 5 μL of FITC-F4/80, APC-CD86, and PE-CD206 for 30 min at 4 °C in the dark. Flow cytometry was used to detect the *in vitro* re-education of M2 macrophages.

### 2.12 Quantification of mRNA by real-time PCR

RAW264.7 cells were subsequently induced to M1 macrophages using IFN-γ (2.5 ng/mL) and LPS (200 ng/mL), and M2 macrophages using IL-4(10 ng/mL). Total RNA was isolated from cells with 400 μL TRIzol reagent. The isolated RNA (1 μg) was reversely transcribed into cDNA with BeyoRT™ II First Strand cDNA Synthesis Kit. QRT-PCR was performed using the PowerUp™ SYBR™ Green Master Mix on PCR systems, according to the manufacturer's instructions. The sequences of primers are listed as below (Table 1). The relative mRNA levels were calculated using the comparative Ct method (ΔCt).

**Table 1:** Primer sequences for qRT-PCR.

Gene	sense primer sequences	antisense primer sequences
CD86	GGTGGCCTTTTTGACACTCTC	TGAGGTAGAGGTAGGAGGATCTT
iNOS	GTTCTCAGCCCAACAATACAAGA	GTGGACGGGTCGATGTCAC
Arg-1	AGGAAAGCTGGTCTGCTGGAA	ATTTGAAAGGAGCTGTCATTAGGG
GAPDH	CCTCGTCCCGTAGACAAAATG	TGAGGTCAATGAAGGGGTCGT

### 2.13 *In vivo* and *ex vivo* imaging with near-infrared fluorescence (NIRF)

Pan02 cells (1 × 10^6^ cells/mouse) were subcutaneously implanted into the right side of BALB/c nude mice. After the tumor nodules reached 80–100 mm^3^, the mice were intravenously injected with DID, DID NVs and DID-SS NVs (10 μg DiD per mouse), respectively. The fluoresce signals was observed using an IVIS® imaging system (PerkinElmer, USA). Following euthanasia of mice after injection at different time, the blood, tumors, liver, kidneys, spleen, heart, and lungs were removed for *ex vivo* imaging, which was then followed by the fluorescent quantitation analysis.

### 2.14 *In vivo* antitumor effect

The tumor-bearing C57BL/6J mice were randomly allocated into six groups (n = 6): saline, TP (0.4 mg/kg, injection via tail vein (i.v.)), Rg3 (3 mg/kg, i.v.), SS (0.16 mg/kg, i.v.), Mix (TP-PEG-SS + Rg3) and NVs (equivalent dose of TP, SS, Rg3, i.v.). The body weight and tumor volume were measured every other day. At the end of the experiment, the blood samples, livers, kidneys, and tumors were converged from the mice for blood biochemical analysis, hematoxylin and eosin (H&E) staining, and immunohistochemistry (IHC) staining, as previously described [7].

### 2.15 Histological examination

Following paraformaldehyde treatment, the kidney, liver, and tumor tissues were decalcified with 10 % EDTA, dehydrated with gradient ethanol, embedded in paraffin and pathological sectioning. The H&E staining slices was observed under an upright microscope. Ki67 and TUNEL expressions in tumor tissue were analysed by immunohistochemistry, iNOS (M1 macrophage) and CD206 (M2 macrophage) in tumor tissues were detected by immunofluorescence labelling [20].

### 2.16 Data analysis

The data were analysed using the SPSS 25.0 and presented as mean ± SD. T-tests or one-way ANOVA were used to compare data. A *P*-value of less than 0.05 was considered as a statistically significant result.

## 3 Result and discussion

### 3.1 Synthesis and characterization of TP-PEG-SS conjugate

**Fig. 1:**
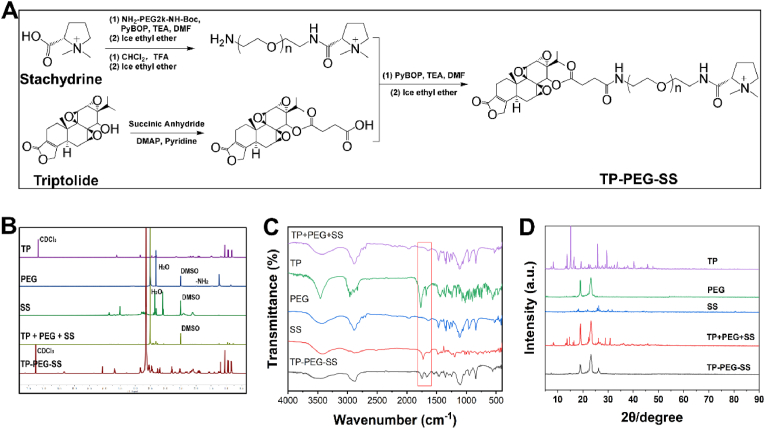
(A) Synthetic route of TP-PEG-SS conjugate; (B) ^1^H NMR spectra, (C) FTIR spectra and (D) XRD diffraction patterns of TP, PEG, SS, and TP-PEG-SS.

To alleviate the systemic toxicity and enhance the activity of TP, we covalently coupled TP with SS by PEG to generate a water-soluble, positively charged TP-PEG-SS conjugate. Three steps were involved in the synthesis procedure of TP-PEG-SS (Fig. 1A): (1) esterification reaction between triptolide (TP) and succinic anhydride, (2) amidation reaction between stachydrine (SS) and Boc-amino-polyethyleneglycol-amino (NH_2_-PEG-NH-Boc, 2 kDa), and (3) the amidation reaction between the reaction products from (1) and (2) gave the final product TP-PEG-SS. The product was characterised using ^1^H NMR, FT-IR and XRD methods. The weight percentage (wt%) of TP and SS in TP-PEG-SS was gauged by HPLC, which were 14.38 % for TP and 5.75 % for SS. The findings indicated that TP and SS were effectively grafted with NH_2_-PEG-NH_2_.

The ^1^H NMR spectra of TP-PEG-SS revealed a new peak at 2.76 ppm (succinic triptolide) and a disappeared peak at 2.0 ppm (NH_2_-PEG-NH_2_), while the physical mixture showed a simple superposition of the peaks of succinic TP, PEG, and SS (Fig. 1B, Fig. S1A). The FT-IR spectra of TP-PEG-SS exhibited a new strong peak of absorption at 1678 cm^−1^, which was attributed to the C=O stretching vibration of its two amide groups. The strong absorption at 1749 cm^−1^, corresponding to the unsaturated lactone group of TP-PEG-SS, caused a blue shift and substantial reduction as compared with TP, indicating a successful introduce of the amide groups (Fig. 1C, Fig. S1B). PEG showed two sharp peaks at 19° and 23°, whereas TP and SS displayed many sharp peaks at the 2θ range of 7-50°, indicating that PEG, TP and SS had some degree of crystallinity (Fig. 1D). The physical mixture showed a simple superposition of the XRD peaks of PEG, TP and SS. TP-PEG-SS had three XRD peaks of at 18.86°, 23.26° and 26.28°, reflecting the reduced crystallinity of TP and SS as well as a slight change in PEG. The results showed that TP and SS were successfully grafted onto PEG to produce TP-PEG-SS.

### 3.2 Preparation and characterization of NVs

**Fig. 2:**
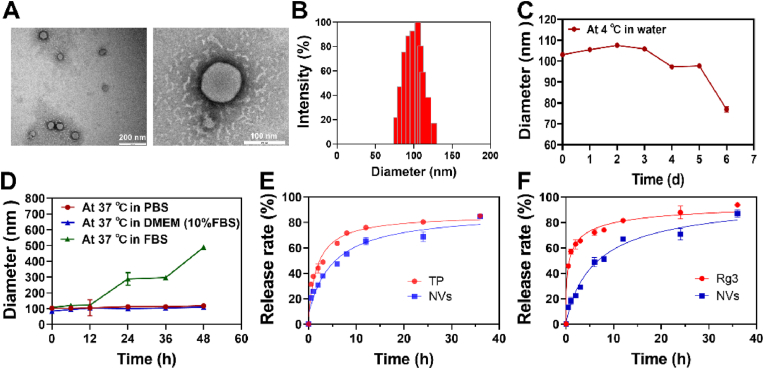
(A) The TEM micrographs of NVs; (B) The particle size distribution of NVs determined by DLS; Stability of NVs measured by DLS (C) in water at 4 °C, and (D) in PBS, 10 % FBS of cell culture medium, or FBS at 37 °C; The TP (E) and Rg3 (F) release profiles from NVs in PBS at 37 °C.

**Fig. 3:**
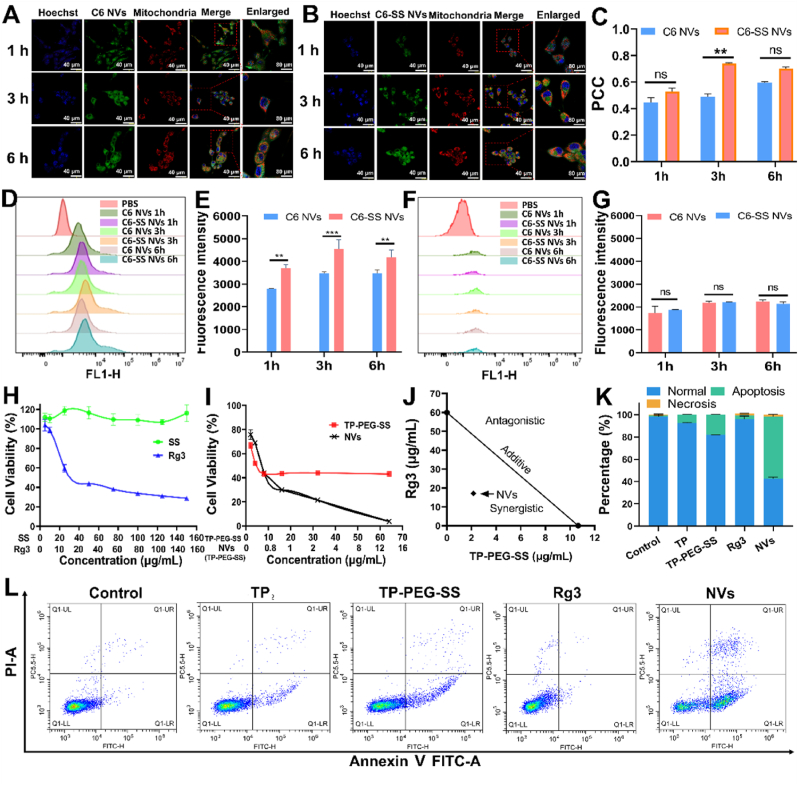
Laser confocal microscopy images of Pan02 cells cultured with (A) C6 NVs or (B) C6-SS NVs for 1 h. Blue: Hoechst 33342; Red: Mito-Tracker Red CMXRos; Green: Coumarin 6. Scale bars: 40 μm. (C) PCC analysis for detection of colocalization regions. (D) Flow cytometry analysis of M2 macrophages for various nanovesicle uptake. (E) Cellular uptake efficiency of C6 NVs and C6-SS NVs. (F) Flow cytometry analysis of M1 macrophages for various nanovesicle uptake. (G) Cellular uptake efficiency of C6 NVs and C6-SS NVs. The cell viability rates of pancreatic cancer cells after treatment with (H) SS and Rg3, (I) TP-PEG-SS and NVs (equiv. TP-PEG-SS). (J) Isobologram analysis of the synergistic inhibitory effects of TP-PEG-SS and Rg3 on Pan02 cells. (K) Quantitative analysis of apoptotic Pan02 cells. (L) Apoptosis of Pan02 cells after treatment with saline, TP, TP-PEG-SS, Rg3 and NVs for 24 h ∗∗*P* < 0.01, ∗∗∗*P* < 0.001, compared with the C6 NVs group.

The nanovesicles (NVs) were prepared by self-assembly of the amphiphilic TP-PEG-SS conjugate, ginsenoside Rg3 and lecithin. In the process, the weight ratios of the different ingredients were screened based on the vesicle size and synergistic anticancer efficacy. As shown in Table S1, a weight ratio of lecithin (PC) to Rg3 at 3:1 can form stable blank nanoparticles with the size of lower than 100 nm and negative zeta potential. The formulation was further optimized by incorporating TP-PEG-SS into the blank nanoparticles. The particles at a high ratio (weight ratio >10:1) of Rg3 and TP-PEG-SS were less than 50 nm, whereas those formed at a ratio below 5:1 had a particle size of less than 100 nm and low zeta potential (Table S2). The bulky hydrophilic groups of TP-PEG-SS and Rg3 may occupy space on the particle surface, changing the phospholipids' arrangement, compressing the NVs, and reducing the particle size, which explains the difference of those particle sizes. Rg3 and TP-PEG-SS have an optimal ratio of 15:2, which falls within the range of their weight ratios for the best anticancer activity (Fig. 3J). The NVs were characterised on the basis of their morphology and size distribution using TEM and DLS. As illustrated in Fig. 2A and B, the NVs display a spherical morphology with a diameter of approximately 100 nm, which is substantially consistent with the particle size detected by DLS. The NVs had a mean particle size of 119.17 ± 1.34 nm with a narrow size distribution of 0.153 ± 0.019 (PDI) and zeta potential of 12.40 ± 1.76 mV, indicating a positively charged nanoparticle. The NVs had superior encapsulation efficiency of 86.21 ± 1.61 % for TP-PEG-SS and 91.30 ± 2.46 % for Rg3, and drug loading capacity of 2.78 ± 0.05 % for TP-PEG-SS and 22.09 ± 0.59 % for Rg3, respectively.

The *in vitro* stability of NVs was investigated by DLS in different conditions. As shown in Fig. 2C–D and Fig. S2A–F, the particle size of NVs in water was decreased from 103.05 nm to 79.46 nm at 4 °C within seven days. When NVs were stored in PBS and DMEM containing 10 % FBS at 37 °C, the particle sizes were observed to remain 100 nm for a period of 48 h, demonstrating the relative stability of NVs. However, the particle size of NVs in FBS was increased markedly to ∼300 nm within 24 h, followed by a platform period of 12 h. The findings indicated that NVs were able to remain stable in water at 4 °C for 5 days, and in PBS and DMEM containing 10 % FBS at 37 °C for 48 h. Whereas, NVs in FBS were stable during the first 12 h and the second platform period.

The release of TP and Rg3 from NVs were measured by using a dialysis bag. Fig. 2E and F revealed that the free TP and Rg3 were rapidly exuded from the dialysis bag within 6 h, while those were released continuously from NVs within 12 h. The release rate of free TP and Rg3 were 48.91 % and 65.65 % at 3 h, followed by a sustained increase (84.74 % and 93.78 % at 36 h). Simultaneously, free Rg3 was slightly easier to elute from the dialysis bag than free TP in PBS. The drug release profiles of NVs showed a similar release trend for TP and Rg3. The release rate of TP and Rg3 reached 37.92 % and 29.13 % at 3 h, followed by reaching the peaks of 84.74 % and 87 % at 36 h. Compared to the free TP and Rg3, the NVs exhibited a slow and sustained release within 36 h. The findings indicated that NVs could maintain a stable drug concentration in blood, which would significantly reduce the frequency and dosage of administration.

### 3.3 Double-targeted and double cellular uptake of NVs

To investigate the tumor mitochondrial-targeting capacity of NVs, the coumarin 6 (C6), Rg3 and TP-PEG-SS were co-loaded into nanovesicles to generate C6-SS NVs. In addition, C6 was loaded into nanoparticles without Rg3 and TP-PEG-SS to generate C6 NVs. Hoechst33342 was used to stain the cell nucleus blue. The mitochondrion was stained red with Mito Tracker Red CMXRos. The co-localisation of tracers and mitochondria was observed under a laser confocal microscope. As shown in Fig. 3A and B, the fluorescence signals of C6-SS NVs and C6 NVs in mitochondria increased in a time-dependent manner. Moreover, the fluorescence intensities of C6-SS NVs in mitochondria were around 1.3 times stronger than those of C6 NVs for each time point (*P* < 0.0001, Fig. S3). The Pearson coefficients showed 53 %, 74 % and 70 % of colocalization regions for C6-SS NVs at 1, 3 and 6 h, while 44.67 %, 49 % and 59.67 % for C6 NVs (Fig. 3C). SS-TP LPs, a stachydrine-modified cationic liposome we previously reported, can actively target mitochondria in pancreatic cancer cells via electrostatic interaction, and then reduce the mitochondrial membrane potential [7]. Thus, C6-SS NVs might be actively transported into tumor cells via the mitochondria pathway. It can therefore be concluded that NVs have a strong mitochondrial-targeting capacity, which may be attributed to the grafted stachydrine with a positive charge.

The macrophages in the tumor are mainly of M2-like TAMs, with few M1 macrophages. The M2 and M1 phenotype macrophages can be analysed by detection of Arg-1 and iNOS gene expression using PCR. In our studies, the PCR results exhibited a high Arg-1 expression in M2-like TAMs, a high iNOS expression in M1 macrophages, and both low Arg-1 and iNOS levels in RAW264.7 cells (Fig. S4). The cellular uptake of C6-SS NVs in M2-like TAMs was evaluated by flow cytometry. As shown in Fig. 3D and E, the fluorescence intensity in C6-SS NVs groups was significantly higher than that in C6 NVs groups at different time (*P* < 0.01, *P* < 0.001). The findings indicated that C6-SS NVs containing Rg3 bore higher accumulation in M2-like TAMs, compared with C6 NVs without Rg3. It may be attributed to the high expression of the glucose transporter GLUT-1 on M2-like TAMs [20,22]. GLUT-1 can regulate the transport of Rg3 containing two glucose residues [20], so C6-SS NVs might be transported into M2-like TAMs via the glucose transporter GLUT-1. Therefore, NVs can specifically target M2-like TAMs via Rg3.

Furthermore, the cellular uptake of C6-SS NVs was also evaluated in M1 macrophages. The results showed that the fluorescence intensity in C6-SS NVs groups was very low as same as that in C6 NVs groups at different time (*P* > 0.05, Fig. 3F and G), indicating that C6-SS NVs had difficulty being transported into M1 macrophages. In summary, NVs has double-targeted capability to target tumor cells and M2 macrophages, which will greatly improve accumulation of NVs in the tumor regions and enhance the antitumor effect with a low toxicity.

### 3.4 *In vitro* cytotoxicity

Previous studies had demonstrated that TP and Rg3 are effective anti-tumor ingredients extracted from traditional Chinese medicine [29,30]. However, the highly toxic TP often seriously damages healthy tissues and organs during treatment of pancreatic cancer. Accordingly, we designed and synthetized a cationic prodrug, TP-PEG-SS, which was anticipated to enhance the tumor-targeting capacity and reduce cytotoxicity of TP without compromising its anti-tumour efficacy. In nanovesicles, Rg3 was used to further enhance anticancer efficacy of TP-PEG-SS and regulate the tumor microenvironment to reduce adverse effects caused by TP. To confirm this hypothesis, the CCK-8 approach was employed to evaluate the bioactivity of NVs on Pan02 cells. As shown in Fig. 3H and I, NVs, TP-PEG-SS, and Rg3 exhibited a pronounced inhibition effect on Pan02 cells, while stachydrine had negligible cell inhibition capability. The 50 % inhibiting concentrations (IC_50_) of NVs, TP-PEG-SS, and Rg3 were 2.15 ± 0.37 μg/mL (eqv. TP of 0.3096 μg/mL), 10.66 ± 1.51 μg/mL (eqv. TP of 1.53 μg/mL), and 59.96 ± 3.61 μg/mL, respectively. The NVs showed nearly 5 times tumor inhibition capacity of TP-PEG-SS, and more than 28-fold tumor inhibition capacity of Rg3, which might be attributed to the double-targeted accumulation of NVs in Pan02 cells and M2 macrophages, and the synergistic antitumor effects of Rg3 and TP-PEG-SS. The isobologram method was employed to depict their synergistic antitumor effects (Fig. 3J). In the coordinate system, the additive inhibition effects of TP-PEG-SS and Rg3 were represented by the points on the diagonal line, the antagonistic inhibition effects located above the line and synergistic inhibition effects located below the line. As shown in Fig. 3J, the antitumor effect of NVs was represented by the point situated at the bottom left of the contour plot, indicating a significantly enhanced synergistic tumor-inhibitory efficacy. The findings showed that the NVs effectively inhibited the growth of tumors through enhancing the double-targeted capability and the synergistic antitumor effect of TP-PEG-SS and Rg3.

NVs entered the tumor mitochondria and elicited apoptosis of Pan02 cells, which were evaluated by flow cytometry (Fig. 3K–L, Fig. S7). For the TP, TP-PEG-SS, Rg3 and NVs groups, the apoptosis rates were 7.34 %, 18.06 %, 3.18 % and 56.02 %, respectively. The NVs showed over 3 times apoptosis rate than TP-PEG-SS, and nearly 18-fold apoptosis rate than Rg3, which were essentially consistent with their tumor inhibition capability. The results indicated that the low apoptosis induction rate of Rg3 on tumor cells resulted in a low inhibition rate, the effect of TP-PEG-SS was moderate, while NVs significantly improved the apoptotic efficiency and antitumor effect due to the nanoscale effect, the mitochondrial-targeting capacity, and the synergistic effect of Rg3 and TP-PEG-SS on tumor cells [7,15,26]. These findings further elucidated that NVs efficiently inhibited the Pan02 cell growth through the apoptosis-mediated antitumor pathway.

### 3.5 M2 phenotype macrophage re-education of NVs

**Fig. 4:**
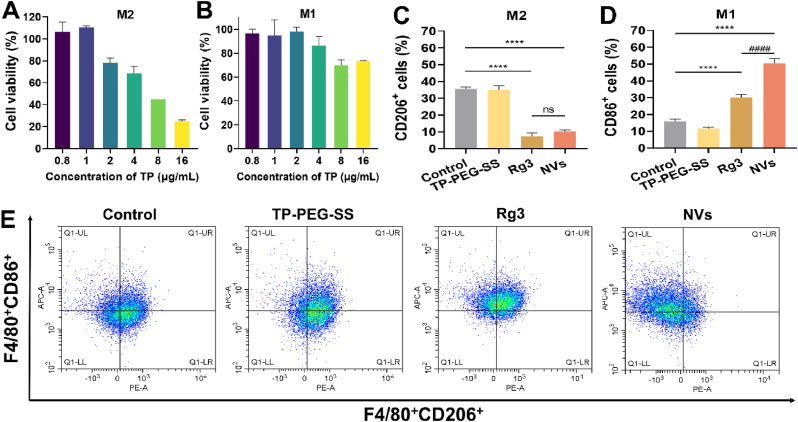
The cell viability of (A) M2 macrophages and (B) M1 macrophages after treatment with NVs. The percentages of macrophage populations with specific macrophage markers (C) M2-hypotype (F4/80^+^CD206^+^) and (D) M1-hypotype (F4/80^+^CD86^+^) were analysed by flow cytometry. (E) The flow cytometry detection of the “re-education” of M2 macrophage after treatment with saline, TP-PEG-SS, Rg3 and NVs for 24 h ∗∗∗∗*P* < 0.0001, compared with the Control group; ^####^*P* < 0.0001, compared with the Rg3 group.

NVs exhibited potent growth inhibition of Pan02 cells. However, whether NVs can reduce the M2-like TAMs by reprogramming M2 macrophages to remodel the immunosuppressive tumor microenvironment, which requires further investigation. As shown in Fig. 4A and B, the IC_50_ of NVs in M2 and M1 macrophages were 7.19 ± 0.17 μg/mL (eqv. TP of 1.04 μg/mL) and 38.95 ± 5.37 μg/mL (eqv. TP of 5.61 μg/mL). Obviously, the inhibition capacity of NVs to M2 macrophages was over 5 times greater than that to M1 macrophages, which might play a positive role in regulating the tumor immune microenvironment.

In the tumor microenvironment, tumor cells continuously recruit macrophages to the tumor site, and continuously release cytokines to transform macrophages into M2-like TAMs, thereby promoting tumor growth [31]. Inspiringly, the M2-like TAMs can be re-educated into M1 macrophages by drug stimulation, thus exerting anti-tumor effects. Flow cytometry was used to analyse the *in vitro* “re-education” of M2 macrophages after treatment with PBS, TP-PEG-SS, Rg3, and NVs. As shown in Fig. 4C–E and Fig. S8, there was no significant difference in the percentage of M1 or M2 macrophages between the TP-PEG-SS group and the control group (*P* > 0.05). The percentage of M2 macrophages (F4/80^+^CD206^+^) was significantly decreased after treatment with Rg3 and NVs (Fig. 4B). However, the percentage of M1 macrophages (F4/80^+^CD86^+^) was significantly increased in the Rg3 and NVs groups (Fig. 4C). In addition, the NVs group showed significantly higher percentage of M1 macrophages than the Rg3 group (*P* < 0.001). Those findings indicated that it may be attributed to the potent “re-education” capability of Rg3 on M2 macrophages.

To confirm this hypothesis, Rg3 was used to regulate the polarization of M2 macrophages (Arg-1) towards M1 macrophages (iNOS, CD86), which was evaluated using PCR. The results showed that Rg3 significantly reduced the Arg-1 expression, suggesting a pronounced inhibition effect on M2 macrophages (*P* < 0.01, Fig. S5A). Whereas, Rg3 also enhanced significantly both iNOS and CD86 expression levels (*P* < 0.01, Fig. S5B–C), indicating that Rg3 can “re-educate” M2 macrophages into M1 macrophages. Consequently, in addition to its anti-tumor effect, Rg3 endows NVs with another ability to regulate the tumor microenvironment by re-educating M2 into M1 macrophages.

### 3.6 Biodistribution and pharmacokinetic analysis

**Fig. 5:**
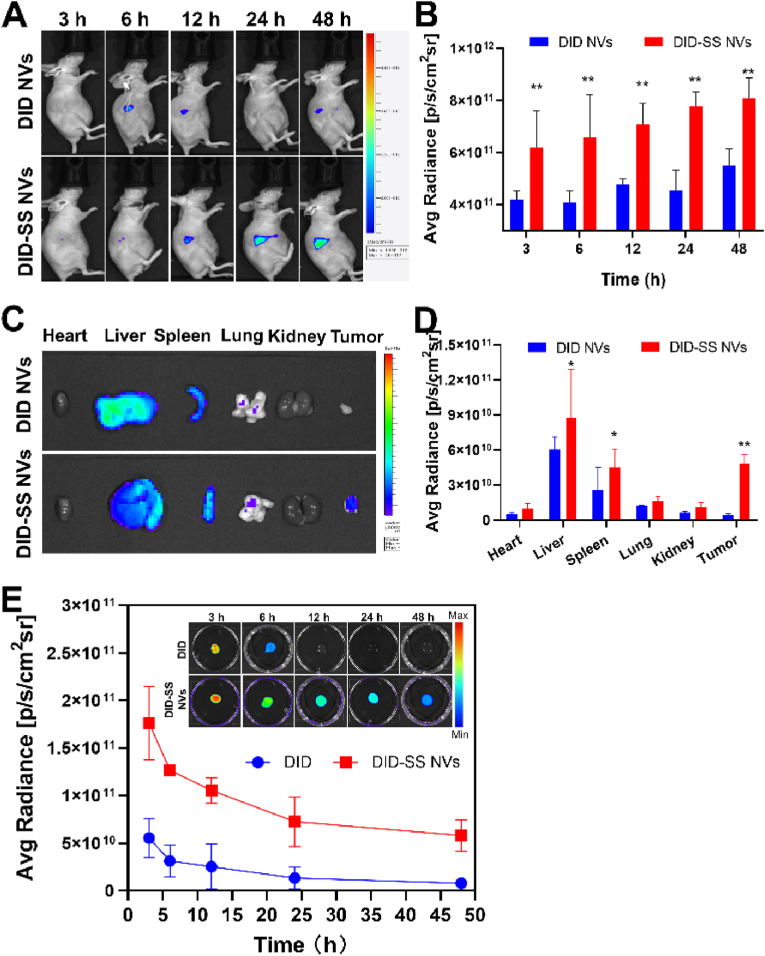
Biodistribution and pharmacokinetics in mice after tail intravenous injection of DID, DID NVs (Non-targeted nanovesicles) or DID-SS NVs (Targeted nanovesicles) at different times. (A) *In vivo* imaging of tumor-bearing mice after intravenous injection of DID NVs and DID-SS NVs at the indicated time points. (B) Quantitative analysis of fluorescence intensity in the tumor region of the tumor-bearing mice. (C) Representative *ex vivo* imaging of hearts, livers, spleens, lungs, kidneys and tumors. (D) Quantitative analysis of fluorescence intensity in the organs and tumors. (E) The blood half-life of DID and DID-SS NVs in mice. ∗*P* < 0.05, ∗∗*P* < 0.01, compared with the DID NVs group (n = 3).

An effective nanoplatform can enhance the enrichment of drugs in tumor regions and reduce TP-induced adverse effects. As shown in Fig. 5 and Fig. S6, the *in vivo* and *ex vivo* NIRF imaging were performed in tumor-bearing mice after intravenous injection of DID NVs and DID-SS NVs by using an IVIS® Lumina III system. The fluorescence of DID-SS NVs group was enriched in the subcutaneous tumor regions at the given time points post-injection, whereas negligible fluorescence was observed in DID NVs group (Fig. 5A). The DID-SS NVs group demonstrated significantly higher fluorescence intensity at the tumor site during the 48-h treatment period, compared with the DID NVs group (*P* < 0.01, Fig. 5B). Especially, the fluorescence intensity of the DID-SS NVs group increased by about 2 times at 24 h, compared with the DID NVs group. The results suggested that DID-SS NVs might accumulate in xenografted tumor regions via tumor mitochondrial-targeting and M2 macrophage GLUT-1-mediated endocytosis. *Ex vivo* NIRF imaging study revealed that the fluorescence was mainly accumulated in livers, spleens and tumors, while very few fluorescence was observed in hearts, lungs and kidneys (Fig. 5C and Fig. S6). The fluorescence intensities in livers, spleens and tumors of DID-SS NVs group were significantly higher than those of DID NVs group (*P* < 0.05, *P* < 0.01). Furthermore, the fluorescence intensity in tumor regions of DID-SS NVs group was 10.6-fold higher than that of DID NVs group at the 24 h (Fig. 5D). The fluorescence intensities of tumor tissues in DID NVs group were 1.10–4.85 times higher than those of spleen, heart, lung and kidney tissues except liver tissues. In summary, DID-SS NVs had a high accumulation in the tumor regions, which might be attributed to the cell uptake of nanovesicles by tumor cells through electrostatic interaction and by M2 macrophages through glucose transporters. The findings can support the potential clinical value of NVs as an effective nanodrug delivery platform for improvement of drug availability and reduction of side effects of TP.

A prolonged blood circulation makes it easier for NVs to sufficiently accumulate in the tumor region. To understand kinetic profile of NVs in blood circulation, the fluorescence intensity of the blood samples was examined to quantify the drug concentration in blood. As illustrated in Fig. 5E, DID was rapidly cleared from the bloodstream, while DID-SS NVs had high blood concentrations and slow elimination from the bloodstream. The blood half-life of DID-SS NVs was 17.75 h, which was over 4 times longer than that of DID (T_1/2_ = 4.18 h). Thus, NVs may be easier to aggregate in tumor sites than DID, because NVs has a longer blood retention time, an enhanced penetration and retention effect (EPR effect) and the double-targeted capability.

### 3.7 *In vivo* antitumor effect and safety

**Fig. 6:**
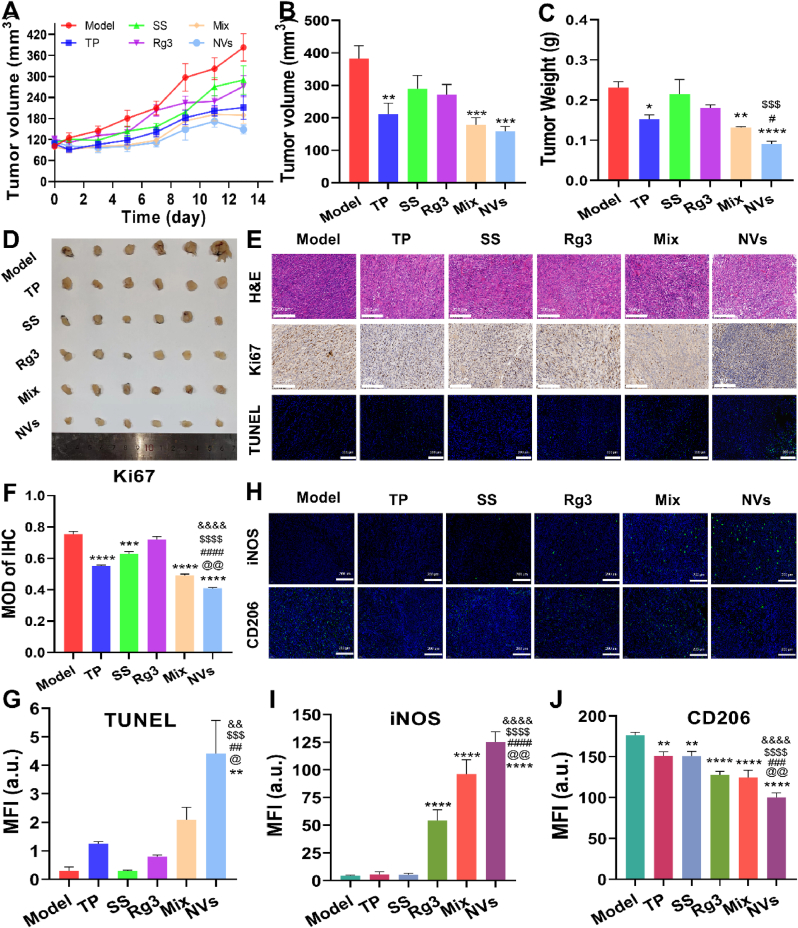
(A) Changes in tumor volume throughout the course of pancreatic cancer treatment. The volume (B) and weight (C) of tumors at the end of the experiment. (D) The solid tumors excised from mice at the end of the experiment. (E) Histological examination of tumor tissues, Ki67 immunohistochemical staining of tumour tissues, and TdT-mediated dUTP Nick-End labelling (TUNEL) immunofluorescence staining of tumor tissues. Semi-quantitative analysis of Ki67 immunohistochemistry (F) and TUNEL immunofluorescence (G). Scale bars: 200 μm. (H) The expression of CD206 (M2 macrophages) and iNOS (M1 macrophages) on tumor tissues. Scale bars: 200 μm. Semi-quantitative analysis of CD206 (I) and iNOS (J) fluorescence intensity. ∗*P* < 0.05, ∗∗*P* < 0.01, ∗∗∗*P* < 0.001, ∗∗∗∗*P* < 0.0001, compared with the Model group; ^&&^*P* < 0.01, ^&&&&^*P* < 0.0001, compared with the TP group; ^$$$^*P* < 0.001, ^$$$$^*P* < 0.0001, compared with the SS group; ^#^*P* < 0.05, ^##^*P* < 0.01, ^###^*P* < 0.001, ^####^*P* < 0.0001, compared with the Rg3 group; ^@^*P* < 0.05, ^@@^*P* < 0.01, compared with the Mix group.

The curative effects of NVs on Pan02 tumor-bearing mice were assessed by monitoring tumor size, tumor weight, and immunohistochemical staining. As shown in Fig. 6A, the NVs group exhibited the smallest change in tumor volume during 13 days, indicating superior antitumor effects as compared with other treatment groups. Upon concluding the experiment, the variation trends of tumor volume and tumor weight were essentially coincident with the *ex vivo* tumors observed in the different groups (Fig. 6B–D). As shown in Fig. 6E, the HE-staining tumor tissue slices in the model group exhibited intact cell morphology, dense and neatly arranged tumor cells, and clear nuclei. In comparison to the model group, the tumor cells in each treatment group demonstrated different degrees of cellular shrinkage, damage and necrosis. The significant shrinkage, cell necrosis and loose structure were observed in the tumor cells of the NVs group, which collectively indicated that NVs had effective antitumor activities. As shown in Fig. 6E–G, the Ki67 and TUNEL immunohistochemical staining were carried out on the tumor tissues. The results demonstrated that the level of Ki67 protein was reduced to different degrees in each treatment group, while the NVs group had a significant down-regulation as compared with other groups (*P* < 0.01, *P* < 0.0001, Fig. 6E and F), indicating that the NVs effectively inhibited the proliferation of tumor cells. Furthermore, the TUNEL results exhibited that the model group and SS group had a negligible green fluorescence signal, whereas the NVs group displayed the largest areas of green fluorescence signals as compared with other groups (*P* < 0.05, *P* < 0.01, *P* < 0.001, Fig. 6E and G). The findings indicated that NVs exhibited the highest anticancer efficacy by inhibiting proliferation of tumor cells and inducing apoptosis of tumor cells.

During tumorigenesis, the macrophages are recruited and subsequently transformed into the M2-like TAMs to facilitate tumor progression. Fortunately, the polarization of tumor-associated macrophages is reversible, and Rg3 can “re-educate” the M2 phenotype into the M1 phenotype [20]. The antitumor M1 macrophages overexpress iNOS, whereas the pro-tumor M2 macrophages overexpress CD206. Based on these markers, M1 and M2 macrophages were determined by immunofluorescence. As shown in Fig. 6H, NVs, mixture and Rg3 could increase the green fluorescence signals of iNOS (M1 biomarker), while reducing the green fluorescence signals of CD206 (M2 biomarker), compared with the model group. Especially, the regulation effects of NVs were greater than those of free TP, SS, Rg3 and mixture, which might be due to the enhanced uptake efficiency via the M2-targeted nanovesicles (*P* < 0.01, *P* < 0.001, *P* < 0.0001, Fig. 6H–J). The findings showed that NVs could ameliorate the immunosuppressive tumor microenvironment to improve anticancer efficacy by transformation of M2-to-M1 phenotype. Hence, the combination of tumor cell apoptosis with M1 macrophage polarization might be a potential strategy for the treatment of pancreatic cancer.

**Fig. 7:**
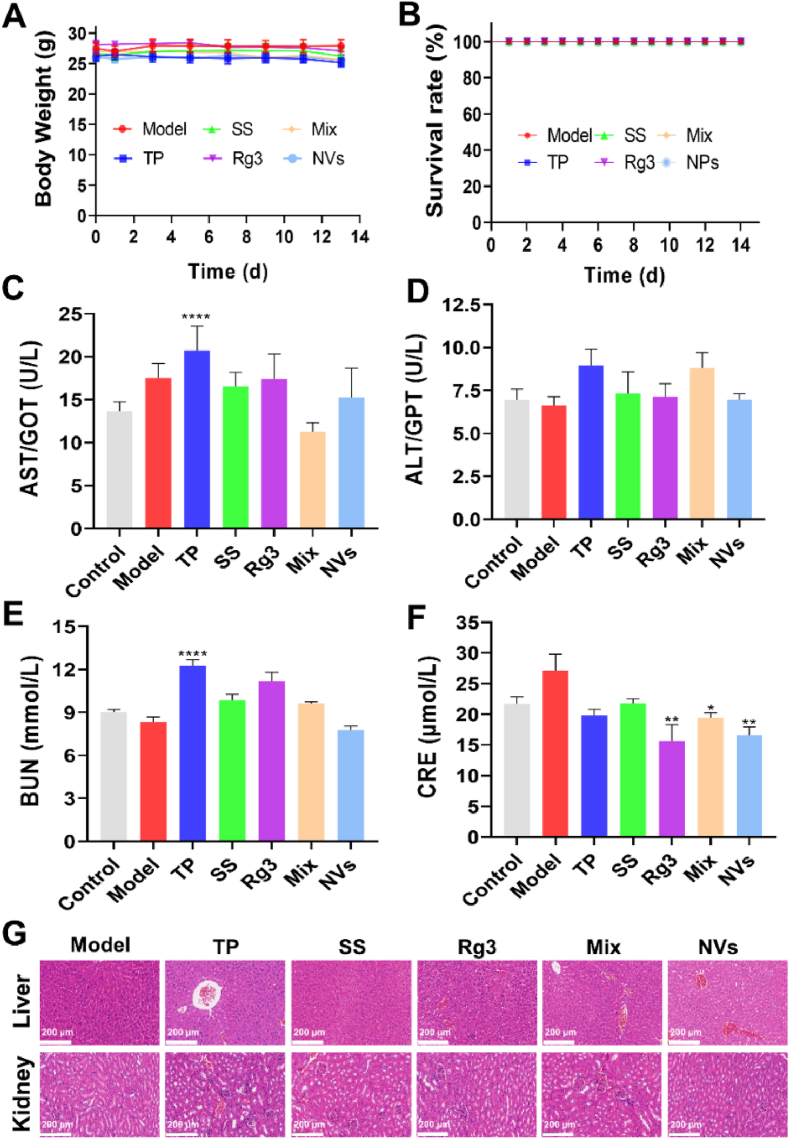
(A) Variations of body weight during the treatment period. (B) Mouse survival rates within two weeks. (C–F) Blood biochemical tests for ALT, AST, BUN, and CRE in mice treated with different drugs. ∗*P* < 0.05, ∗∗*P* < 0.01, ∗∗∗∗*P* < 0.0001, compared with Model group. (G) Histological examination of livers and kidneys in mice. Scale bars: 200 μm.

TP has highly efficient anticancer activity, whereas it also has strong systemic toxicity. As shown in Fig. 7A, except for a minor weight loss observed in the TP group, there was slight difference in body weight of the other groups. This might be attributed to the cytotoxicity of TP [7], whereas the formulations containing Rg3 alleviated the TP-induced systemic toxicity. The toxic risk of NVs in the blood system was evaluated by hemolysis assay. The hemolysis ratio of NVs in red blood cells increased in a dose-dependent manner (Fig. S9). However, all hemolysis ratios were below the hemolysis standard by less than 5 %. There was no hemolysis reaction even at 350 μg/mL of NVs (eqv. TP of 50.43 μg/mL), indicating that the risk of NVs in blood circulation was acceptable. Thus, no mice died during the experiment, suggesting that NVs do not have lethal toxicity (Fig. 7B). To further confirm whether the main organs were damaged by the NVs, liver and kidney functions were detected by using ALT, AST, BUN and CRE assay kits. As shown in Fig. 7C–F, TP caused a significant increase of AST and BUN levels (*P* < 0.0001), whereas NVs, Rg3, and physical mixture notably decreased the CRE levels (*P* < 0.05, *P* < 0.01), compared with those in the model group. However, the NVs group maintained the normal levels of ALT, AST, BUN and CRE. The findings indicated that NVs could reduce the liver and kidney toxicity induced by TP. Furthermore, HE staining showed that the TP group had mild edema and slight cellular damage in the livers and kidneys, whereas the NVs group exhibited no obvious edema or cellular damage (Fig. 7G). In sum, the NVs had effectively reduced the TP-induced systemic toxicity, particularly hepatotoxicity and nephrotoxicity, while enhancing the anticancer efficacy.

## 4 Conclusion

We synthesized a novel TP-PEG-SS prodrug, which exhibited excellent water solubility, mitochondrial targeting capability and high antitumor activity with lower toxicity. Subsequently, a nanovesicle (NVs) containing TP-PEG-SS and ginsenoside Rg3 was prepared to enhance synergistic antitumor efficacy and reduce the TP-induced cytotoxicity. The NVs were selectively accumulated in tumor mitochondria and M2 macrophages, followed by inducing apoptosis of tumor and transformation of M2-to-M1 phenotype. It was further confirmed that NVs could accurately target the tumor regions and improve the immunosuppressive tumor microenvironment in tumor-bearing mice, thereby exerting a stronger antitumor effect and lower systemic toxicity than TP-PEG-SS and free TP. Our studies suggest that NVs may be a promising anticancer nanovesicle for dual-targeted therapy in pancreatic cancer.

## CRediT authorship contribution statement

**Jiaxing Wang:** Writing – original draft, Methodology, Formal analysis, Data curation. **Jingru Cui:** Investigation, Data curation. **Yujie Chen:** Investigation, Formal analysis. **Huijie Zhou:** Methodology, Investigation. **Xiaofang Li:** Validation, Resources, Funding acquisition. **Xiangxiang Wu:** Writing – review & editing, Funding acquisition, Conceptualization. **Rongyi Zhou:** Writing – review & editing, Supervision, Funding acquisition. **Huahui Zeng:** Writing – review & editing, Supervision, Resources, Conceptualization.

## Ethical approval statement

We conducted the animal experiments for the evaluation of the antitumor efficacy of NVs which is indispensable for new drug discovery. All mice were housed in a pathogen-free ventilated room (25 ± 1 °C, 55–65 % humidity, 12 h light-dark cycle) and furnished with water and food. At the end of experiment, the mice were euthanized with CO_2_. In the hemolysis assay, the anesthesia, blood sample collection and euthanasia of the small animals were consistent with ethical permission. All animal experiments were conducted in accordance with the guidelines of the Institute's Animal Care (GB/T35892-2018), and approved by the Experimental Animal Ethics Committee of Henan University of Chinese Medicine (Approval ID: IACUC-202308023).

## Declaration of competing interest

The authors declare that they have no known competing financial interests or personal relationships that could have appeared to influence the work reported in this paper.

## Data Availability

Data will be made available on request.
