# Supplementary material for: Self-assembled triptolide prodrug nanovesicles loading with ginsenoside Rg3 for double-targeted therapy of pancreatic cancer
Source: Mater Today Bio. 2025 Jun 18;33:102004. doi: 10.1016/j.mtbio.2025.102004 (PMC12221477; doi:10.1016/j.mtbio.2025.102004)
Supplement: Multimedia component 1 [file mmc1.docx]

**Supplemental Information**

**Self-assembled triptolide prodrug nanovesicles loading with ginsenoside Rg3 for double-targeted therapy of pancreatic cancer**


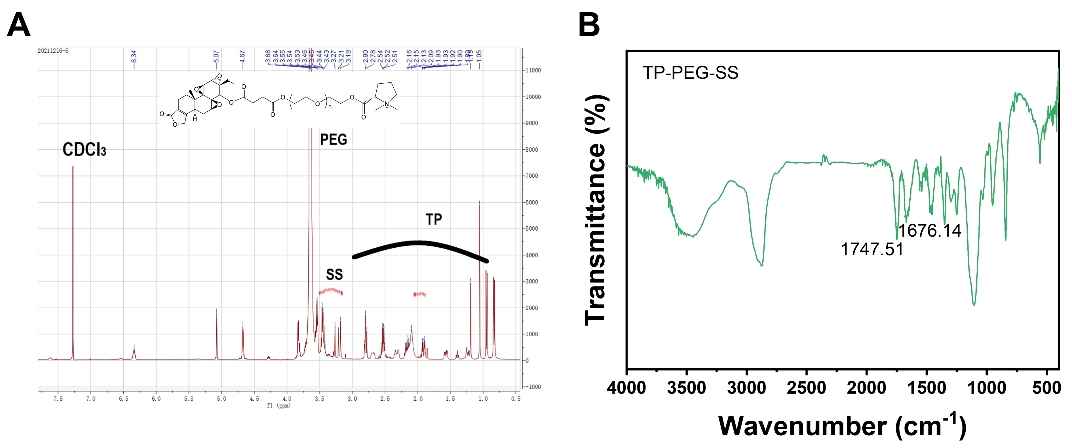


Figure S1. (A) ^1^H-NMR spectra and (B) FTIR spectra of TP-PEG-SS conjugate.

Table S1. Sizes and zeta potential of Rg3 NVs (without TP-PEG-SS) at different weight ratio between lecithin and ginsenoside Rg3). (Mean ± SD, n=3).

| weight ratio (Lecithin:Rg3) | particle size (nm) | PDI | zeta potential (mV) |
| --- | --- | --- | --- |
| 2:1 | 139.37±1.62 | 0.224±0.019 | -22.43±2.73 |
| 3:1 | 94.23±1.30 | 0.201±0.010 | -23.20±0.57 |
| 4:1 | 183.15±2.92 | 0.274±0.001 | -19.61±1.25 |
| 5:1 | 135.72±1.14 | 0.294±0.015 | -15.17±0.33 |

Table S2. Sizes and zeta potential of NVs at different weight ratio between ginsenoside Rg3 and TP-PEG-SS, based on a ratio of lecithin (PC) to Rg3 at 3:1. (Mean ± SD, n=3).

| weight ratio  (Rg3:TP-PEG-SS) | particle size (nm) | PDI | zeta potential(mV) |
| --- | --- | --- | --- |
| 1.25:1 | 95.48±0.69 | 0.161±0.002 | -5.85±0.26 |
| 2.5:1 | 77.14±0.24 | 0.181±0.004 | 3.13±0.50 |
| 5:1 | 82.61±0.66 | 0.215±0.002 | 5.04±1.92 |
| 7.5:1 | 119.17±1.34 | 0.153±0.019 | 12.40±1.76 |
| 10:1 | 50.47±0.22 | 0.210±0.003 | -1.87±3.02 |


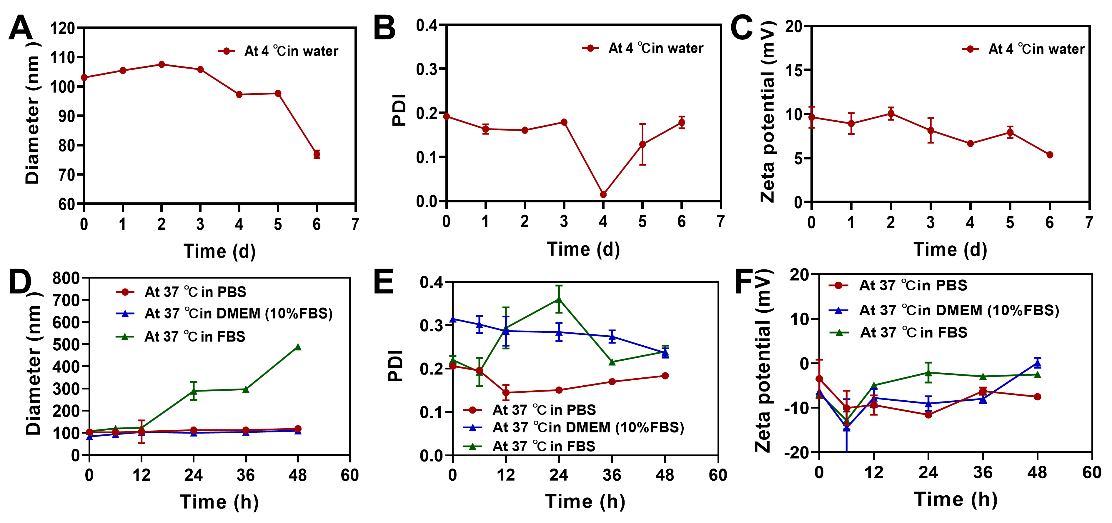


Figure S2. *In vitro* stability of NVs measured by DLS (A-C) in water at 4°C, and (D-F) in PBS, 10% FBS of cell culture medium, or FBS at 37 °C.


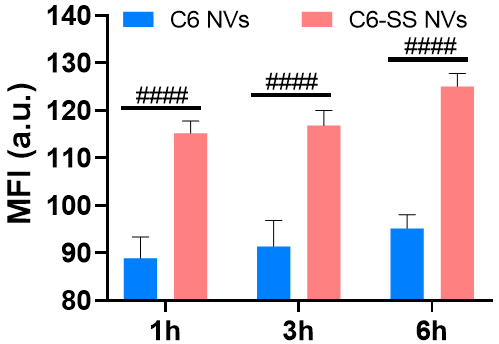


Figure S3. Quantitative analysis of fluorescence intensity of the colocalization regions in tumor mitochondria. ^####^*P* < 0.0001, compared with the C6 NVs group.


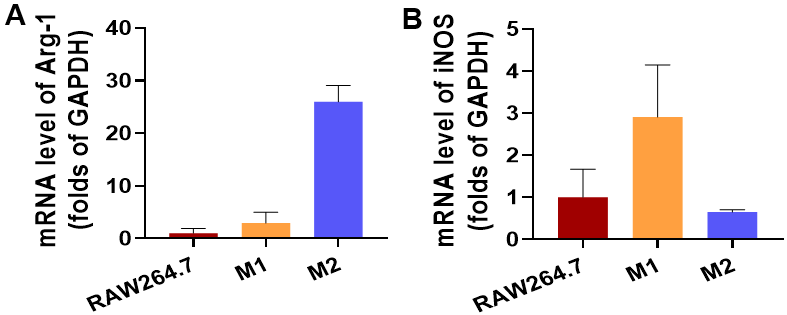


Figure S4. The phenotypes of macrophages were analyzed by detection of iNOS and Arg-1 gene expression using PCR.


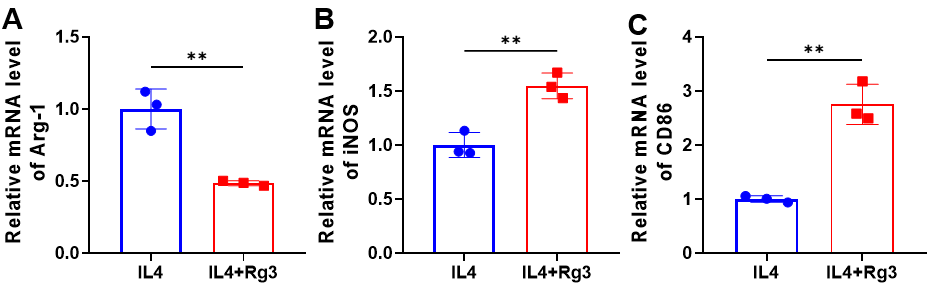


Figure S5. Rg3 regulates the polarization of M2 macrophages towards M1 macrophages, which were evaluated by detection of CD86, iNOS and Arg-1 gene expression using PCR. ***P* < 0.01, compared with the M2 macrophage group.


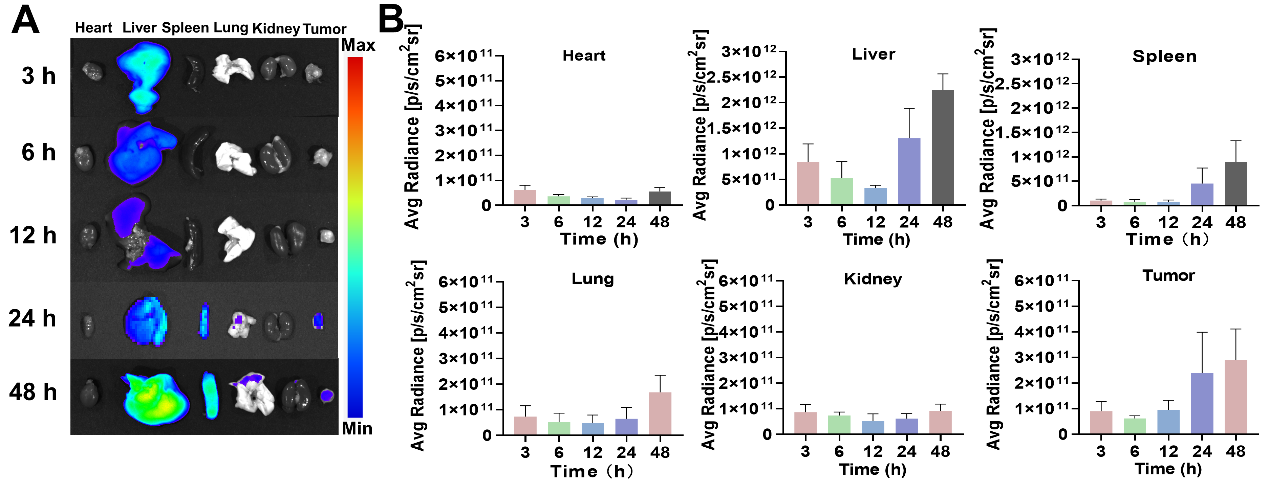


Figure S6. Biodistribution in Pan02 tumor-bearing mice after tail intravenous injection of DID-SS NVs (n = 3). (A) *Ex vivo* imaging of hearts, livers, lungs, kidneys, spleens and tumors at different time and (B) their corresponding fluorescence intensity analysis.
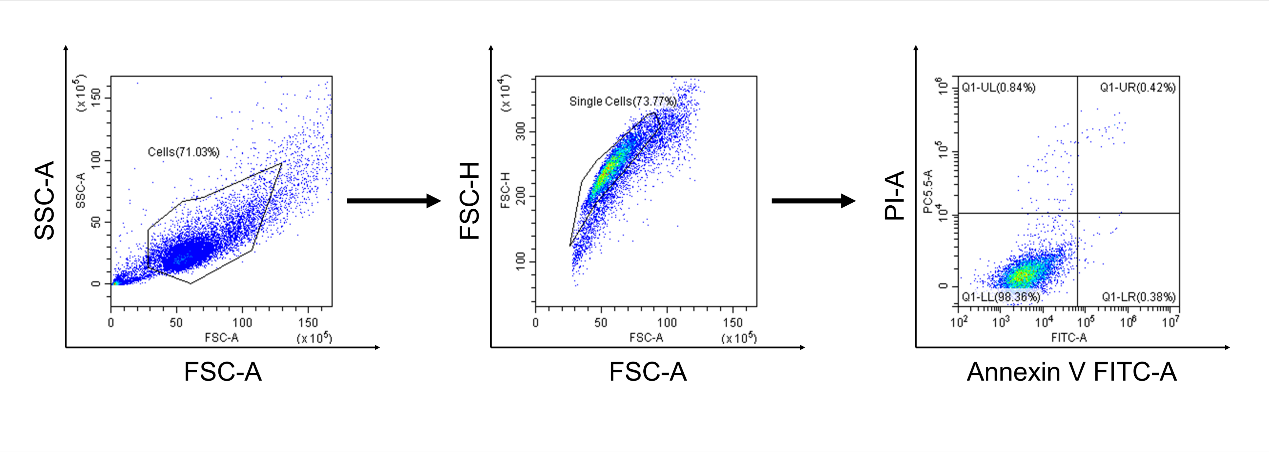


Figure S7. Flow cytometry gating strategy for the apoptosis analysis in Pan02 cells.


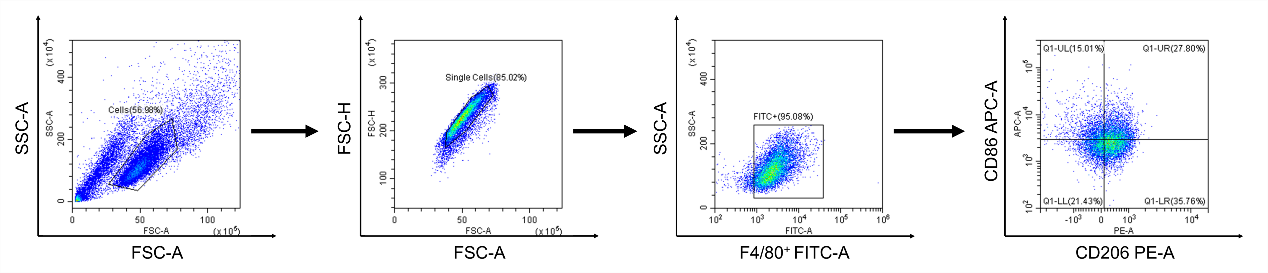


Figure S8. Flow cytometry gating strategy for the analysis of M2-like macrophages and M1-like macrophages.


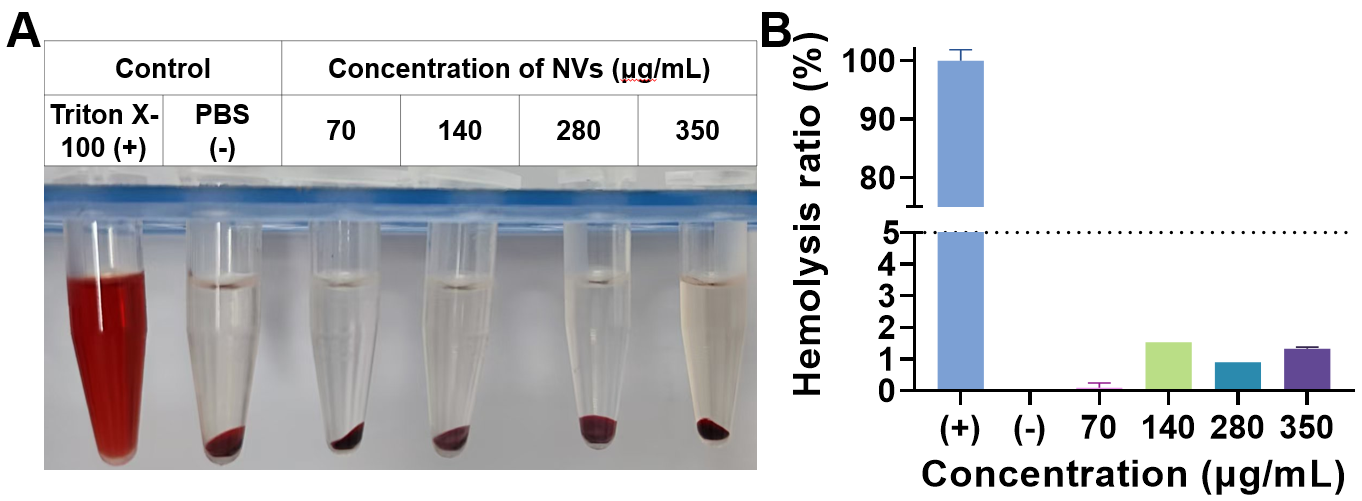


Figure S9. Hemolysis assays of red blood cells treated with NVs, Triton-X, and PBS. (A) Photograph of hemolysis of red blood cells after the treatment with different concentrations of NVs. Triton X-100 and PBS are used as positive (+) and negative (-) controls, respectively. (B) Hemolysis percentage of red blood cells exposed to varying concentrations of NVs. Hemolysis of less than 5% is regarded as harmless. (n = 3).

***In vitro*** **hemolysis test**: The blood sample was collected from the retro-orbital vein plexus of mouse and loaded into a tube containing heparin anticoagulant. After centrifugation, the red cells were collected and resuspended in PBS, followed by treating red cell suspension (900 μL) with 100 μL of PBS, Triton X-100, NVs for 1 h at 37 °C. After centrifugation, the supernatant was collected and detected by a microplate reader. The hemolysis ratio was calculated as follows:

$$Haemolysis ratio (\%)=\frac{A(sample)-A(-)}{A(+)-A(-)}\times100\%$$

The absorbance of the supernatant incubated with sample, PBS and Triton X-100 were represented with A(sample), A(-) and A(+), respectively.
